# Successful treatment of lower lip venous lake using CO_2_ fractional laser therapy

**DOI:** 10.1097/MD.0000000000047188

**Published:** 2026-04-03

**Authors:** Sedra Abu Ghedda, Bushra Karkour, Mouina Shami

**Affiliations:** a University of Aleppo, Faculty of Medicine, Aleppo, Outside Canada, Syria; b Department of Dermatology and Venereology, Aleppo University Hospital, Aleppo, Outside Canada, Syria.

**Keywords:** CO_2_ fractional laser, vascular abnormalities, venous lake

## Abstract

**Rationale:**

Venous lakes (VL) are common vascular anomalies, typically affecting individuals over 40 and appearing on the lips or oral mucosa. Conventional treatments: such as surgical excision and coagulation, can lead to postoperative complications, especially in cosmetically sensitive areas. Various laser therapies have been proposed; however, some are not readily available in developing countries. This case report explores fractional Carbon dioxide laser (CO_2_ laser) therapy as a safe, minimally invasive alternative with favorable aesthetic and clinical outcomes.

**Patient concerns:**

An 18-year-old Syrian female presented with a 1.8 cm violet, sessile nodule on her lower lip. Although asymptomatic, the lesion caused aesthetic distress and occasional trauma from her teeth.

**Diagnoses:**

Clinical evaluation confirmed the diagnosis of a VL on the lower lip.

**Interventions:**

The patient underwent 3 sessions of fractional CO_2_ laser therapy targeting the lesion.

**Outcomes:**

Complete healing was achieved without tissue loss, scarring, or recurrence during an 8-month follow-up period. No adverse effects were reported throughout the treatment process.

**Lessons:**

Fractional CO_2_ laser therapy shows promise as an effective and minimally invasive treatment for VL, particularly in cosmetically sensitive areas. Further prospective studies are needed to validate its efficacy and compare outcomes across different treatment modalities.

## 1. Introduction

Venous lakes (VL) are prevalent vascular anomalies characterized by the dilation of small veins in the upper dermis. These formations consist of a delicate layer of endothelial cells, reinforced by a robust fibrous wall.^[1]^ Histological analysis reveals that the vascular lakes possess extremely thin walls, composed primarily of a single layer of endothelial cells with minimal muscle content and scant supporting fibrous tissue. These lakes are extensively connected to small veins. In a limited number of specimens, small arteries appear to supply the lakes, although in most cases no arterial input is identified.^[2]^ They are often observed in nearly 50% of people over the age of 40, specifically in the lips and oral mucosa.^[2]^ They can appear in multiple forms and range in size from 5 to 15 mm in diameter.^[3]^ The diagnosis is primarily clinical and relies on the observation of lesion compression, which leads to ischemia in the affected area, eliminating the necessity for an oral biopsy.^[4]^ They are often asymptomatic, thus treatment for VL is sought for cosmetic purposes or due to frequent bleeding. Traditional approaches for managing vascular lesions include techniques like surgical removal, cryotherapy, infrared coagulation, sclerotherapy, and electrocoagulation.^[5,6]^ While these methods have proven effective, they are not without risks. Patients may experience various postoperative issues such as bleeding, swelling, discomfort, alterations in skin texture, scarring, or allergic responses to the treatments administered.^[7]^ Laser and light-based therapies have emerged as effective treatments for VL, with pulsed dye lasers demonstrating significant efficacy.^[8]^ However, it is often inaccessible in many developing countries due to their high costs. The Nd:YAG laser has also proven effective, particularly for small lesions measuring 6 mm or less.^[9]^ There have been few published reports on the use of carbon dioxide lasers (CO_2_ lasers) for treating VL, as this method is generally not advised for vascular lesions.^[4,10]^ In this study, we present a successful case of a lower lip VL treated with 3 sessions of CO_2_ fractional laser, which occurred without any complications.

## 2. Case presentation

An 18-year-old female presented to the outpatient dermatology clinic with a lesion on her lower lip.

**Figure 1.**
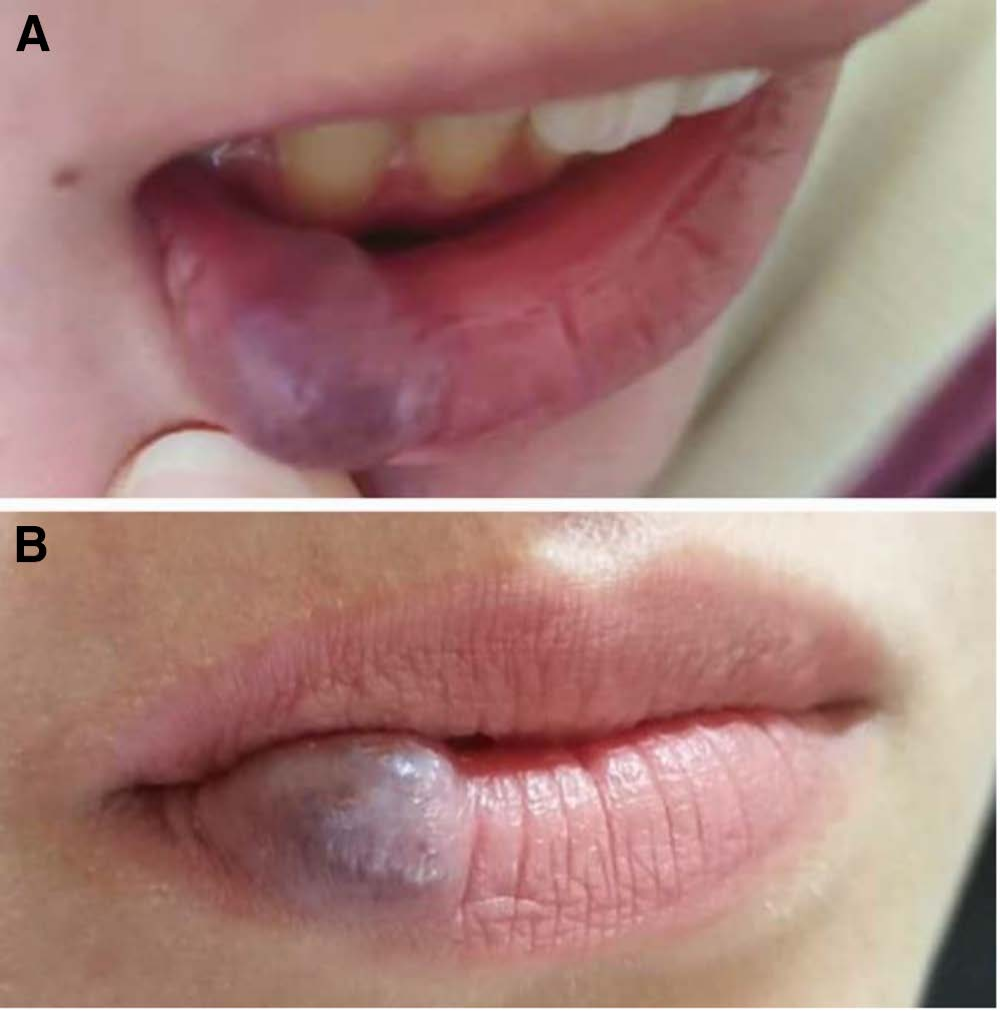
(A) and (B) Venous lake lesion before treatment.

**Figure 2.**
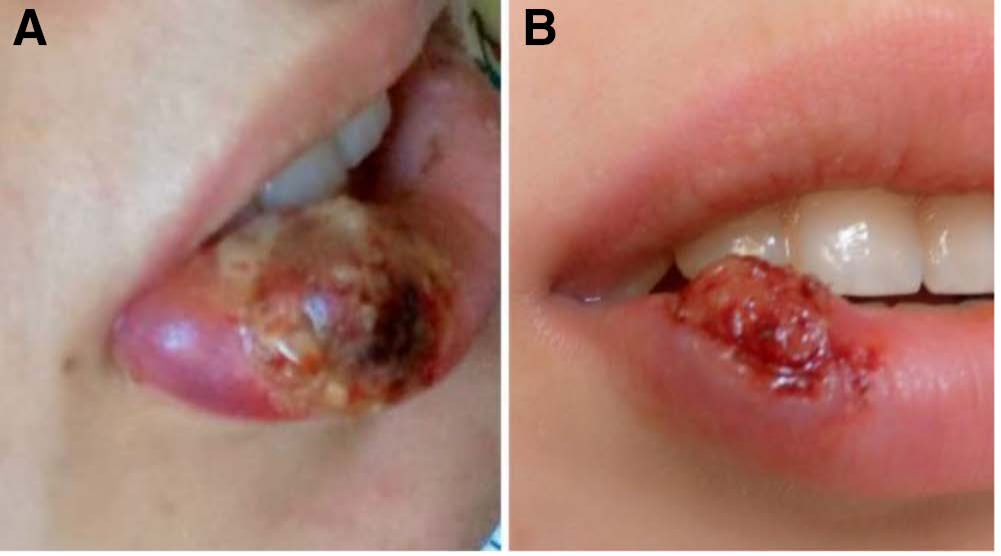
(A) Picture of the lesion immediately after the first session showing edema bruising and pinpoint bleeding. (B) Picture of the lesion 3 weeks after the first session showing granulation tissue formation.

**Figure 3.**
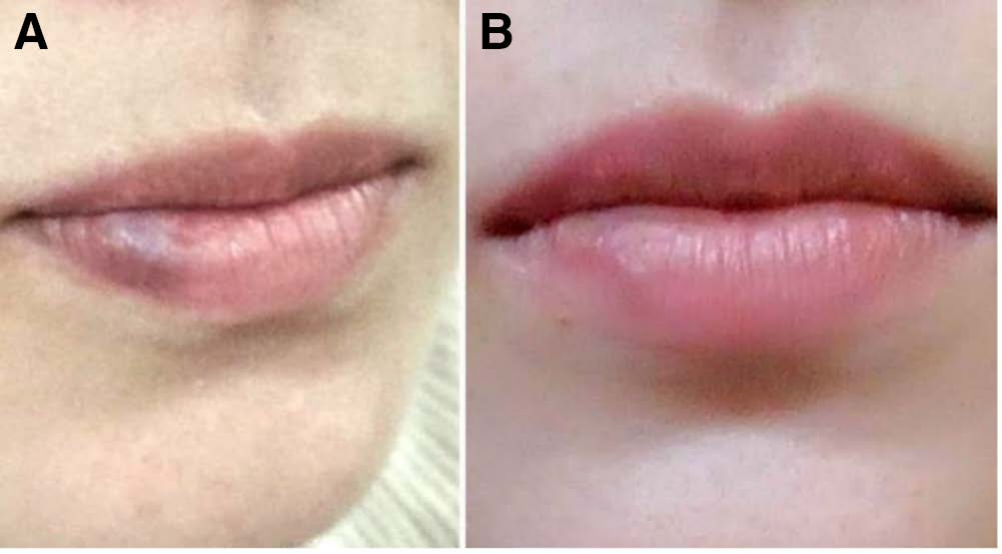
(A) Picture of the lesion after the second treatment showing noticeable improvement. (B) Picture of the lesion after the last session showing complete resolution without any tissue loss.

Upon examination of the lips, a violet sessile nodule measuring 1.8 cm was observed on the right side of the lower lip’s vermilion Figure 1. The lesion had a smooth surface and exhibited vascular characteristics confirmed by diascopy. The diagnosis of VL was confirmed. Although it was not painful, its prominence on the patient’s face led to aesthetic concerns. Additionally, the lesion occasionally experienced trauma from her teeth, resulting in temporary episodes of swelling. The lesion was asymptomatic and had been present for 3 years without prior treatment. A CO_2_ laser (10,600 nm, Ex Matrix, Sincoheren, Korea) was utilized following local anesthesia with Lidocaine 2%. The fractional laser treatment created microscopic treatment zones within the lip lesion, facilitating collagen remodeling and targeting blood vessels through selective photothermolysis. Employing a superpulsed mode, the laser emitted high-energy, ultra-short pulses, which reduced collateral thermal necrosis by approximately 50% compared to continuous wave CO_2_ lasers, resulting in faster healing and minimized scarring. The specific laser parameters included an energy setting of 10 mJ per microbeam, a density of 80%, and 2 passes with superpulsed pulse duration. The procedure concluded after confirming hemostasis through simple pressure applied to the lesion site. She underwent 3 sessions of fractional CO_2_ laser treatment, spaced 2 months apart Figure 2 and 3. The treatment resulted in complete healing of the lesion, with no loss of lip tissue and no recurrence observed during an 8-month follow-up after the final session Figure 3. The patient experienced no side effects or complications throughout the treatment process.

## 3. Discussion

The CO_2_ laser is typically employed for treating photo-aging and mild scarring, rather than for vascular lesions.^[11]^ Nevertheless, previous studies have demonstrated its effectiveness in addressing vascular lesions, including pyogenic granulomas.^[12]^ In this study, we utilized a CO_2_ laser to treat VL on the lower lip due to the unavailability of pulsed dye lasers.

A study conducted by Del Pozo et al evaluated the effectiveness of continuous wave CO_2_ laser treatment in 32 patients with VL, encompassing a total of 34 lesions ranging in diameter from 2 to 10 mm. Remarkably, 31 of the lesions completely resolved after a single session of laser treatment, with no reported complications. Only 1 patient experienced a relapse following the initial session and subsequently required a second treatment.^[4]^ This aligns with our findings, as the lesion was successfully treated without recurrence. However, in our study, we opted for 3 treatment sessions instead of 1 due to the larger size of the lesion. This approach was taken to minimize the risk of tissue loss and complications. In a study conducted by Majamaa et al, similar improvements were observed in patients with VL, with no reported cases of recurrence using continuous wave CO_2_ laser.^[10]^In a multicenter retrospective study involving 143 patients with VL or capillary hemangiomas on the lips, various laser treatments were compared, including Er,Cr:YSGG laser, diode laser, CO_2_ laser, and Nd:YAG laser. The findings indicated no significant differences in outcomes among the different laser modalities. However, both the CO_2_ laser and the Er,Cr:YSGG laser demonstrated greater efficacy, with the added benefit of a zero recurrence rate compared to the other lasers.^[13]^

This suggests that the CO_2_ laser may be effective in treating vascular lesions, which contrasts with the commonly held perception of its limitations in this area.

Fractional CO_2_ laser therapy presents a less aggressive alternative for treating vascular lesions, delivering comparable efficacy to traditional full-field CO_2_ lasers while significantly reducing recovery time. Both modalities are ablative in nature; however, the fractional approach creates microthermal treatment zones, preserving surrounding tissue, and thereby accelerating the healing process. In contrast, conventional CO₂ lasers ablate entire layers of skin, making them more suitable for deeper or more complex lesions, albeit with a prolonged downtime. While earlier studies have predominantly utilized continuous wave CO_2_ lasers, our use of the fractional technique offers a safer profile with enhanced patient tolerance and reduced risk of adverse effects.^[14]^

This study demonstrates the benefits of CO_2_ fractional laser treatment for removing VL, showing no side effects and achieving results in just a few sessions. This makes it a cost-effective option for patients in developing countries.

This study is limited to a single case report, which provides low-level evidence. The absence of a comparison group weakens our evidence and limits the validity of our findings, making it challenging to determine whether the observed outcomes are truly attributable to the intervention. Our review of the literature revealed no direct comparisons between CO_2_ lasers and pulsed dye lasers, indicating a significant gap in current research. Future studies are needed to assess both clinical outcomes and cost-effectiveness, especially in developing countries where access to pulsed dye lasers is limited.

In conclusion, our study highlights the effectiveness of CO_2_ lasers in treating VL on the lower lip, suggesting they can serve as an alternative to pulsed dye lasers. Despite the successful resolution of lesions after multiple sessions, further research with larger, prospective studies is needed to strengthen the evidence base and compare CO_2_ lasers with other treatment modalities. This will enhance our understanding of their role in treating vascular lesions and may expand their clinical applications.

## Author contributions

**Conceptualization:** Sedra Abu Ghedda, Bushra Karkour.

**Data curation:** Sedra Abu Ghedda.

**Investigation:** Sedra Abu Ghedda, Mouina Shami.

**Methodology:** Sedra Abu Ghedda.

**Writing – original draft:** Sedra Abu Ghedda.

**Writing – review & editing:** Sedra Abu Ghedda, Bushra Karkour, Mouina Shami.
